# Cultural Adaptation in Physical Therapy: A Scoping Review of Empirical Evidence and Practices

**DOI:** 10.3390/healthcare14193317

**Published:** 2026-10-05

**Authors:** Jennifer J. Esala, Ilona Fricker, Laura Pizer Gueron, Jepkemoi J. Kibet, Sarah Lawrence, Sara Pearce, Ida Tchuisseu Fonkoue

**Affiliations:** 1 The Center for Victims of Torture, Saint Paul, MN 55114, USA; 2 Divisions of Physical Therapy and Rehabilitation Science, Department of Family Medicine and Community Health, University of Minnesota Medical School, Minneapolis, MN 55455, USA

**Keywords:** cultural adaptation, physical therapy, migrant health, cross-cultural healthcare, scoping review

## Abstract

**Background and Purpose:** The cultural adaptation of physical therapy interventions has been widely recommended, yet the literature remains limited, heterogeneous, and unevenly reported. This scoping review maps the literature on culturally adapted physical therapy and identifies adaptation strategies, key characteristics of the evidence base, and gaps relevant to education and practice. **Methods:** A scoping review of PubMed, Google Scholar, and Elicit (2000–2026) identified 37 peer-reviewed articles on the cultural adaptation of physical therapy. Searches were supplemented by backward and forward citation tracking. Guided by PRISMA-ScR reporting standards, data were charted on study design, article type, populations, outcomes, and reported cultural adaptation approaches. **Results:** Thirty-seven articles were identified, including experimental, non-experimental quantitative, qualitative, and non-empirical sources. The literature was published primarily after 2017 and reflected substantial heterogeneity in populations, intervention types, and outcomes. Five experimental studies were identified, which assessed patient activation, attendance, adherence, pain-related suffering, pain severity, physical function, and health literacy. Reported findings were mixed, with some studies noting improvements in implementation-related or patient-reported outcomes and others reporting no significant differences across clinical outcomes. Additional evidence came from eight non-experimental quantitative studies and twelve qualitative studies. Adaptation strategies included language, content, and intervention adaptation; addressing norms, values, and context considerations; reconciling explanatory models of health and illness; engaging cultural experts; and training for providers. **Discussion and Conclusions:** This scoping review found that the literature on culturally adapted physical therapy is limited, methodologically heterogeneous, and uneven across intervention types and populations. There is a need for methodologically robust and systematically reported research to clarify adaptation approaches, inform education and practice, and support the development of adaptation frameworks. Strengthening this evidence base may help inform future education policy, clinical guidance, and culturally adapted physical therapy care.

## 1. Background and Purpose

The United States (U.S.) is becoming more culturally and linguistically diverse (CALD). Based on census estimates, nearly half of the U.S. population does not identify as White [1], and one in five speaks a language other than English at home [2]. Since the establishment of the federal Refugee Resettlement Program in 1980, more than three million refugees have been resettled in the U.S., which is more than any other country [3]. CALD populations experience higher rates of chronic health conditions, including those central to physical therapy, such as pain, mobility issues, and other musculoskeletal concerns [4,5,6,7,8]. At the same time, CALD populations are less likely to receive adequate care [8]. There are many factors that limit successful engagement with healthcare among CALD populations, such as limited resources, language barriers, divergent cultural beliefs, and systemic racism [9,10].

The cultural adaptation of interventions is one approach to improving care in CALD populations that has received attention across disciplines. Cultural adaptation is the systematic modification of interventions to consider language, culture, and context to make them increasingly compatible with the patient’s cultural beliefs, understandings, behaviors, and related access barriers [11,12,13]. Researchers argue that healthcare interventions may fail to achieve their full potential in CALD populations due to a lack of cultural adaptation, contributing to persistent health disparities [13]. Still, the specific cultural adaptation strategies required to maintain the effectiveness of adapted interventions remain unknown [14,15,16,17]. Research on the effectiveness of cultural adaptation has yielded variable results, with studies alternately showing reductions, no change, or improvements in efficacy [18]. Further, it is estimated that <1% of published research on culturally adapted interventions specifies cultural adaptation methods [19]. This makes it impossible to identify which elements of a cultural adaptation process are effective.

While research on cultural adaptation is ongoing and draws heavily from psychology and public health [13,16,18,20,21,22,23], specific implications for physical therapy remain largely unexplored. This is surprising given the ways in which physical therapy intersects with cultural considerations. Physical therapy involves physical contact, takes place in a variety of medical and non-medical settings, and requires active participation from patients, all of which require cultural considerations. There is a need to map the literature on the cultural adaptation of physical therapy interventions to inform physical therapy education and practice and, in turn, reduce healthcare inequities for chronic physical health conditions among CALD populations. To address this, we conducted a scoping review of the literature on culturally adapted physical therapy, including study types, populations, interventions, outcomes, and cultural adaptation practices.

## 2. Methods

### 2.1. Articles

This scoping review examined the literature on the cultural adaptation of physical therapy interventions. The review was retrospectively registered with INPLASY (registration no. 202670110). Eligible studies were peer-reviewed articles published in English from 2000 onward that examined how cultural factors informed or shaped adaptations to physical therapy interventions. Publications on the topic of cultural adaptation began increasing in 2000, which is the reason for its selection as the start date for this scoping review (see Graph of Supplementary Materials). Articles were eligible if they described, informed, developed, implemented, or evaluated culturally adapted physical therapy.

Cultural adaptation was broadly defined as modifying healthcare interventions or services to address cultural or linguistic factors. Adaptations could involve intervention content or delivery, communication and language, therapeutic goals, patient-provider interactions, family or community involvement, culturally relevant beliefs and practices, or other contextual factors intended to enhance the relevance, acceptability, accessibility, responsiveness, or effectiveness of care. Studies focused solely on adaptations related to age, gender, sexuality, race, socioeconomic status, rurality, or disability were excluded unless they also incorporated cultural adaptation.

Studies were also eligible if they examined culturally adapted physical therapy or components commonly incorporated into physical therapy practice, including exercise and physical activity, pain education, manual or soft-tissue techniques, rehabilitation, goal setting, and patient or family engagement. These studies had to situate these components explicitly within physical therapy or demonstrate implications for physical therapy care. Multidisciplinary rehabilitation studies were included when physical therapy was an identifiable and substantial component of the intervention and the cultural adaptations or findings applied to physical therapy practice.

Given the emerging nature of this literature, a broad range of publication types was eligible, including quantitative, qualitative, and mixed-methods studies; case reports and case series; and theoretical, conceptual, formative, or exploratory articles with substantive implications for physical therapy practice. Empirical studies using any methodological approach and reporting any outcome were included.

Articles were excluded if they were published before 2000, were unavailable in English, or were study protocols. Studies focused primarily on the cultural or linguistic adaptation of measurement instruments were excluded because this literature is extensive, spans diverse measurement domains, and involves methodological issues distinct from intervention adaptation, including measurement validity, cross-cultural equivalence, and psychometric performance.

Cardiac and pulmonary rehabilitation programs were excluded because they typically integrate medical, behavioral, educational, nutritional, and exercise components, making the specific contribution of physical therapy difficult to isolate. Occupational therapy-only interventions were excluded. Standalone exercise, physical activity, sport, fitness, dance, yoga, movement, or lifestyle interventions were excluded unless explicitly presented as components of physical therapy. Articles focused primarily on physical therapy education, workforce diversity, or general cultural competence were excluded unless the educational or training content directly addressed the adaptation or delivery of physical therapy interventions or clinical care.

Systematic, scoping, narrative, and other literature reviews were excluded from the final evidence synthesis. However, relevant reviews were retained for reference-list and citation searching and to assess the comprehensiveness of the search strategy. Conceptual and theoretical articles remained eligible if they provided original, substantive guidance for culturally adapting physical therapy practice.

### 2.2. Search Strategy

PubMed, Google Scholar, and Elicit were searched in September 2026 to identify the literature on the cultural adaptation of physical therapy. Research suggests that PubMed provides comprehensive access to the biomedical and rehabilitation literature, while Google Scholar broadens retrieval to interdisciplinary journals, which combined support the efficient identification of the relevant literature [24]. Retrieval performance in reviews demonstrates that Google Scholar contributes unique references when added to core biomedical databases such as PubMed [25]. Elicit, an AI-assisted research tool, was used to identify relevant studies that may not have been retrieved through PubMed and Google Scholar [26]. Elicit uses semantic search to identify articles based on conceptual similarity to a research question, allowing the identification of the relevant literature that may use different terminology.

PubMed was searched using a combination of terms representing two core concepts: (1) physical therapy interventions and (2) cultural adaptation. Terms within each concept were combined with OR, and the two concepts were combined with AND. The final search strategy was as follows:

(physiotherap* OR “physical therapy” OR “physical therapist*” OR “manual therap*” OR bodywork OR “soft tissue therap*” OR “trigger point therap*” OR massage OR “Physical Therapy Modalities” [Mesh] OR “Physical Therapy Specialty” [Mesh] OR “Massage” [Mesh]) AND (“cultur* adapt*” OR “cultur* tailor*” OR “cultur* competen*” OR “cultur* sensitiv*” OR “cultur* responsiv*” OR “cultur* appropriate*” OR “cultur* congruen*” OR “cultur* inform*” OR “cultur* ground*” OR “cultur* relevan*” OR “cultur* safe*” OR “cultur* modif*” OR “Cultural Competency” [Mesh]).

Google Scholar was used as a supplementary search source using the search terms physiotherapy cultural adaptation. Results were screened sequentially in relevance-ranked order until 50 consecutive results failed to identify a potentially eligible study, which served as the prespecified stopping criterion.

In Elicit, “Across any population, condition, country, and delivery setting, what the peer-reviewed literature published from 1 January 2000 through 30 September 2026 describes, develops, implements, evaluates, or synthesizes cultural adaptation, cultural tailoring, cultural responsiveness, cultural safety, or cultural modification of physiotherapy/physical therapy, or highly related therapeutic body-based interventions?” was entered into Elicit on 4 September 2026. One hundred nine relevance-ranked records returned by Elicit were screened using the same eligibility criteria applied to records identified through the other search methods. Records already identified through PubMed, Google Scholar, or citation searching were treated as duplicates, and additional eligible studies (*n* = 2) identified through Elicit were incorporated into the review.

**Figure 1:**
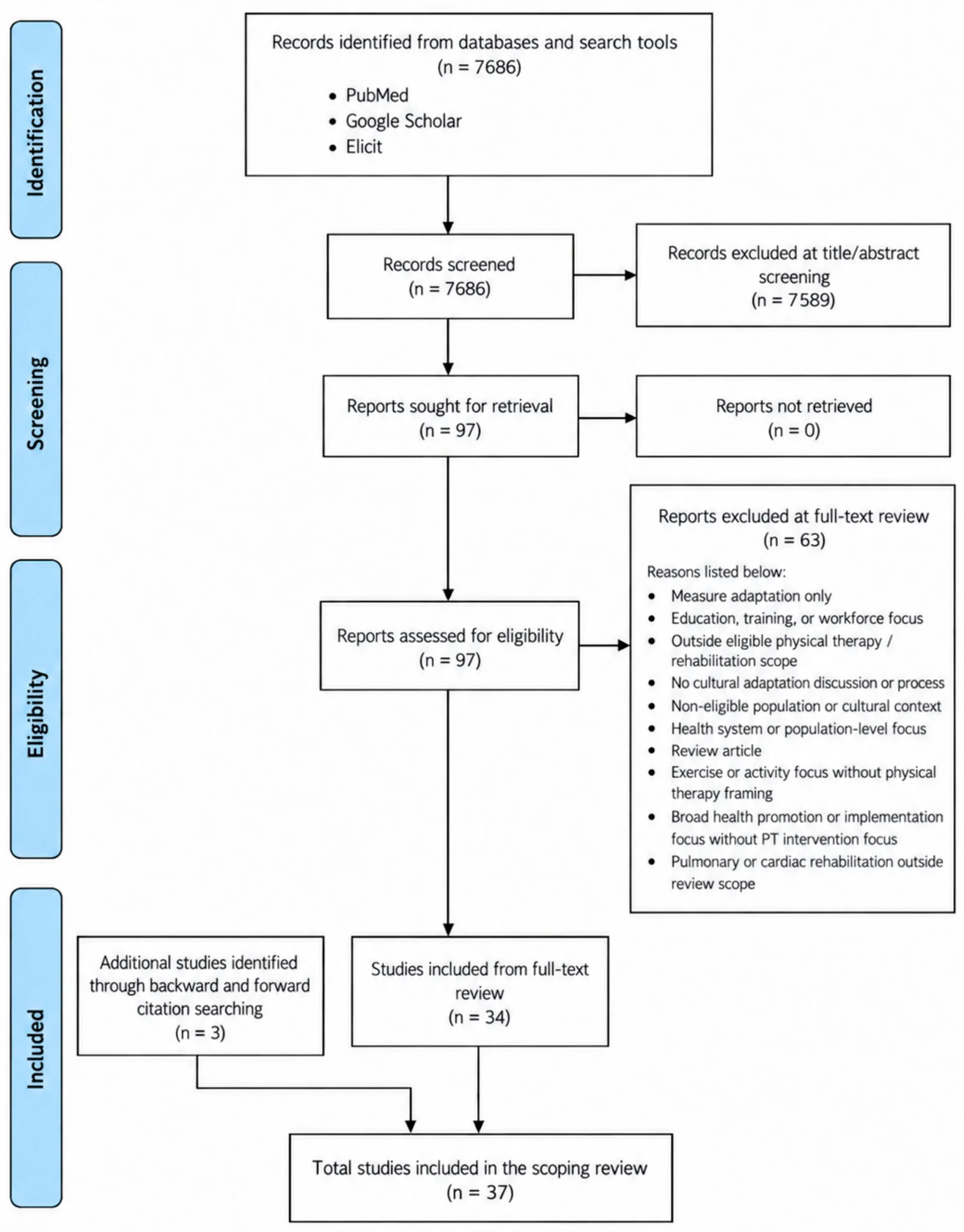
PRISMA flow diagram of study identification and selection.

The search strategy yielded 7686 articles (see Figure 1). This review supplemented database searches with systematic backward and forward citation tracking of all included studies. Following title and abstract screening by two independent reviewers, 97 articles were retained for full-text review. Based on full-text review by three independent reviewers, 37 articles met the inclusion criteria and were included in this review. Disagreements were resolved through discussion and consensus.

Data were charted from the included articles using a standardized data extraction form. Seven reviewers independently extracted data, with each article reviewed by at least two reviewers. The extracted data were compiled and reviewed by one author (JE). Inconsistencies were resolved through discussion and consensus and, when necessary, by cross-checking the original source material. Study authors were contacted when data were missing or incomplete.

Cultural adaptation practices were analyzed using a hybrid deductive–inductive approach. An initial coding framework was developed from the a priori data extraction framework. During preliminary review and coding of the included studies, additional adaptation themes that were not fully represented in the initial framework were identified and incorporated into the coding scheme. Studies were then coded using both the initial and emergent categories. Individual studies could be assigned to multiple adaptation categories when more than one type of cultural adaptation was described. The coding and resulting category structure were reviewed by all authors; disagreements were resolved through discussion and consensus.

Consistent with scoping review methodology, the aim of the review was to map the extent, characteristics, and nature of the literature rather than to assess study quality, appraise risk of bias, or estimate intervention effectiveness; therefore, outcome findings are presented descriptively as reported by study authors.

## 3. Results

Thirty-seven articles met the inclusion criteria for this scoping review. The majority of these articles (25 of 37) were published after 2017. This included five experimentally designed studies, eight non-experimentally designed quantitative studies, twelve qualitative studies, and twelve non-empirical articles. Ten studies used established tools or processes to guide cultural adaptation, and some used multiple, including ADAPTE [27], the Barrera cultural adaptation model [28], Beaton cross-cultural adaptation guidelines [29], Campinha–Bacote’s model [30], ecological validity model (EVM) [28], intervention mapping [31,32], Purnell model for cultural competence [33], RE-AIM framework [34], social ecological model [35], and Two-Eyed Seeing framework [36]. Results from the empirical articles are presented first, followed by a thematic summary of cultural adaptation strategies identified in all article types.

### 3.1. Empirical Articles

There were five experimentally designed articles on culturally adapted physical therapy (see Table 1). One of the studies utilized a waitlist comparator [37], which does not provide information about the effect of cultural adaptation on physical therapy interventions. We summarize the four other experimentally designed studies to characterize the outcomes assessed and summarize the range of reported findings related to culturally adapted physical therapy interventions.

The largest of the four experimentally designed studies examined written educational materials for low back pain and depression [38]. The study compared standard translated materials (*n* = 148) to culturally sensitive materials (*n* = 161), which were adapted based on the results of focus groups with migrant participants. Culturally sensitive materials achieved significantly higher patient-perceived usefulness immediately after the intervention. However, six months after receiving the materials, participants reported no significant differences in perceived usefulness. For depression, back pain, and quality of life, there were no significant differences immediately after receiving the material or at the six-month follow-up. The authors acknowledged study limitations, including that the usefulness assessment tool lacked formal validation, outcomes relied on subjective questionnaires, participants were clinically heterogeneous, and moderation analyses were limited by sample size.

**Table 1:** Experimentally designed studies on the cultural adaptation of physical therapy.

Author	Methods	Intervention	Comparator	Results
Brady et al. 2023 [39]	Quasi- experimental design (*n* = 126)	Cultural peer mentoring program for chronic pain (*n* = 55)	Routine care (*n* = 71)	The intervention group showed significantly higher scores on the patient activation measure (PAM), indicating improvements in knowledge of their health condition and confidence managing it. No significant differences for feasibility measures or health literacy.
Brady et al. 2018 [40]	Randomized controlled trial (*n* = 48)	Culturally adapted physiotherapy (*n* = 24)	Usual physiotherapy care (*n* = 24)	The intervention group showed better attendance, adherence, and reduced pain-related suffering. No significant differences for satisfaction, pain severity, interference, physical function, or negative emotional state.
Holzel et al. 2016 [38]	Randomized controlled trial (*n* = 309)	Culture- sensitive adapted materials for depression and low back pain (*n* = 161)	Standard translated materials for depression and low back pain (*n* = 148)	The intervention group showed significantly higher reported usefulness immediately after intervention. No significant differences for usefulness at follow-up for depression, back pain, or quality of life, either immediately after receiving the material or at follow-up.
Orhan et al. 2021 [41]	Randomized controlled trial (*n* = 29)	Culture- sensitive pain neuroscience education (*n*= 15)	Standard pain neuroscience education (*n* = 14)	No significant differences were found for pain knowledge, pain intensity, disability, organic and psychological pain beliefs, pain catastrophization, or fear of movement.
Gamble et al. 2020 [37]	Randomized controlled trial (*n* = 27)	Culturally specific psychotherapy and physical therapy (*n* = 11)	Wait-list (*n* = 16)	While the study is experimentally designed, the waitlist comparison does not allow for an assessment of the influence of cultural adaptation on intervention outcomes. The process for cultural adaptation was also unclear. The intervention group showed significantly reduced persistent pain, anxiety, depression, and post-traumatic stress disorder symptoms.

A quasi-experimental study examined the feasibility of implementing a cultural mentor alongside a pain management and physical rehabilitation intervention [39]. The study compared the pain management and physical rehabilitation program with (*n* = 55) and without (*n* = 71) a cultural mentor. The intervention group achieved significantly higher patient activation. The authors found no significant differences in feasibility of implementation or health literacy. They noted that this study was limited by the use of convenience sampling within a single health district and that the study was underpowered to assess clinical effectiveness.

A pilot study on the impact of integrating culturally informed approaches into physical therapy for chronic pain compared culturally adapted physiotherapy (*n* = 24) to usual physical therapy care (*n* = 24) [40]. The intervention group achieved better attendance, adherence, and pain-related suffering. They found no significant differences in pain severity, interference, physical function, or negative emotional state. The authors pointed to other study limitations, such as adherence data based on self-report and limited generalizability to three ethnocultural communities.

The final experimentally designed study in this review compared culture-sensitive (*n* = 15) and standard (*n* = 14) pain neuroscience education [41]. The study found no significant differences between the groups on pain knowledge, pain intensity, disability, organic and psychological pain beliefs, catastrophizing, or fear of movement. The study was a small pilot trial, and findings should be interpreted descriptively.

Across these studies, reported findings were mixed. The evidence base included few studies with varied interventions, populations, and outcome measures. Outcomes for which individual studies reported improvements in culturally adapted intervention groups included patient activation, attendance, adherence, and pain-related suffering. The perceived usefulness of healthcare materials was greater in culturally adapted treatment groups immediately after the intervention. The outcome areas where culturally adapted interventions did not show greater improvements were health literacy, pain knowledge, pain severity/intensity, pain interference, back pain, physical function, negative emotional state, quality of life, disability, pain beliefs, catastrophizing, and fear of movement. No included studies reported poorer outcomes in culturally adapted intervention groups relative to comparison groups. There were no significant differences in feasibility.

There were eight non-experimentally designed quantitative studies (see Table 2).

One was a descriptive cross-sectional study about nonpharmacological pain therapies preferred by ethnically diverse older adults [43]. They found that while physicians most often recommended exercise and physical therapy for chronic pain management, only exercise was commonly used, and prayer was a common strategy for pain management among African-Americans and Afro-Caribbeans.

The other two studies were pre-post intervention assessments with qualitative components that included patient feedback. For a sports massage course designed for Aboriginal Health Workers in rural Australia, in an anonymous survey, participants suggested that the course was enjoyable, well-organized, useful, and personally relevant [45]. For an intervention focused on osteoarthritis, adapted from Denmark to Canada, they found that 99% of participants reported the program was beneficial; nearly 90% used the information from the program on a daily basis; and over half would be willing to pay >$250 to attend the program [34]. In the same study, they observed comparable pain and function improvements in Canada as in Denmark.

In another study, PNE4Kids was culturally adapted for Turkish children with chronic pain using Beaton’s cross-cultural adaptation guidelines [29]. The adapted materials were evaluated through a three-round modified Delphi study (*n* = 38). Across all rounds, 84% to 100% of experts agreed or strongly agreed regarding the relevance of the content, feasibility of the stories, appropriateness of the visual materials, and clarity of the messages.

Another study developed and evaluated culturally adapted immersive virtual reality (imVR) games for upper-limb stroke rehabilitation in Ethiopia [42]. The intervention was designed to incorporate culturally relevant rehabilitation activities, emphasizing locally meaningful daily activities. Ethiopian therapists and stroke survivors collaborated with international partners to ensure alignment with user preferences and clinical realities. The authors reported that this culturally grounded approach enhanced the clinicians’ (*n* = 4) and stroke survivors’ (*n* = 6) perceived ease of use, usefulness, and acceptance of the intervention.

A pilot pretest–posttest study evaluated the acceptability and preliminary effectiveness of an existing falls-prevention program among 19 individuals poststroke in Guyana [44]. All participants reported enjoying the exercise program, and all completed the 8-week intervention. Following the intervention, 78.9% demonstrated improved walking speed. Fear of falling also decreased, with the proportion of participants reporting high levels of concern about falling declining from baseline to posttest.

Another study examined the feasibility and preliminary effects of adapted traditional Indian folk games on physical activity and related outcomes among 36 individuals with cerebral palsy in India [46]. Participants demonstrated improvements in handgrip strength and skinfold measurements, as well as enhanced physical and emotional quality of life and school functioning.

The final study used a formative mixed-methods approach and intervention mapping to adapt physical therapy for Latino individuals with chronic spine pain [32]. An organizational needs assessment survey (*n* = 358) identified a lack of culturally tailored physical therapy interventions for this population and found that Latino patients were less than half as likely as non-Hispanic White patients to adhere to physical therapy referrals. In response, the authors developed GOALS/Metas, a culturally adapted intervention designed to address the specific needs and experiences of Latino patients with spine pain. Preliminary evaluation using a case series design included intervention completers (*n* = 4) and matched comparators (*n* = 4) receiving standard physical therapy. Participants who received GOALS/Metas demonstrated greater reductions in pain, higher self-efficacy, and lower fear avoidance than matched comparators.

There were twelve qualitative studies (see Table 2), which included multiple methods: interviews (*n* = 7), focus groups (*n* = 4), document analysis (*n* = 1), and a Delphi study (*n* = 1), with some studies employing more than one method. Specific considerations for cultural adaptations identified in the qualitative research included emphasis on function, active versus passive strategies, personalized care, family involvement, gender preferences, and incorporating traditional healing. These and other adaptation strategies are described in depth in the following section.

### 3.2. Types of Cultural Adaptation

The remainder of the Section 3 summarizes the forms of cultural adaptation described in all articles in this review (see Table 3 for additional non-empirical articles).

#### 3.2.1. A Minimum Requirement: Language Adaptation

Twenty-seven articles described language adaptation as a cultural adaptation strategy. This involved the translation of materials, guidance on language use, and employing professional interpreters. Researchers described the provision of translated materials and professional interpretation as a minimum requirement for competent healthcare [33,41,42,43,57]. They also detailed considerations for translation. For instance, one article observed that in some cultures it is acceptable to address a patient by their first name, but in others it is offensive and can negatively impact the patient-provider relationship [29]. Often language adaptation is technical. For example, in a study on an osteoarthritis intervention adapted from Denmark to Canada, “paracetamol” was revised to “acetaminophen” with patient comprehension implications [30]. Sometimes there is no direct translation for terminology. For instance, in a study on parent educational materials on physical therapy, they found that the term “swaddle” does not exist in Spanish [42]. The researchers instead used descriptors of the action of swaddling.

#### 3.2.2. Content Adaptation: Metaphors, Visuals, and Examples

Authors described content adaptation, beyond translation and interpretation, as another cultural adaptation approach. Strategies included using culturally relevant metaphors, visuals, and examples that resonate with the target population. Eight articles described adapting metaphors [27,28,29,30,31,37,50,63], ten described adapting visuals [28,29,31,32,37,38,50,51,54,63], and five described altering examples, wording, or phrases [29,32,48,63].

For instance, one article described content adaptation to Pain Neuroscience Education (PNE) for Turkish patients [50]. They revised functional activity examples to reflect gender roles and common occupations within Turkish society, including cutting vegetables and sewing for women and hammering a nail and playing football for men. PNE also commonly uses the “extra-sensitive alarm” metaphor to explain how a sensitized nervous system can trigger a pain response without ongoing tissue damage. However, one study found that this metaphor did not resonate in the South African context, where alarms were not a familiar or meaningful reference [63]. Instead, the authors adapted the explanation using an anthill metaphor, which was more consistent with participants’ everyday experiences. If you go to the anthill and disturb it, they will try to attack you. If you do this day after day, eventually the ants will attack you for walking near. This represents the cultural adaptation of the “extra-sensitive alarm” metaphor.

In another study on the adaptation of an intervention for Navajo cancer survivors, they named the intervention *Restoring Balance* to reflect the culturally resonant concept of “returning to harmony with the world” [28]. They also incorporated culturally meaningful symbols into the intervention materials: feathers, which signify blessing and healing; twelve dots, which mark the passage of time; and spirals, which symbolize four phases of life.

#### 3.2.3. Addressing Norms and Values

Fourteen studies highlighted the need to adapt physical therapy interventions to address norms and values, such as gender interactions, religious practices, and collectivism [27,30,31,32,35,36,47,48,52,53,54,57,61,62]. For instance, one study conducted with migrant patients underscored that fasting during Ramadan should be accepted by physical therapists unless there are medical reasons against it [47]. They also found that some patients refused mixed-gender pool therapy and noted the importance of offering gender-segregated environments. Another article advised that appointments should be scheduled around prayer times to respect the religious practices of patients [27].

There were also norms around individualism versus collectivism that impacted treatment. Western care tends to emphasize independence, whereas collectivist cultures sometimes emphasize passive approaches, collaborative treatment, and the involvement of family and community. A study with Turkish and Moroccan families found that family members care for the sick by taking over their daily tasks, which clashes with a common therapeutic goal of independence [54]. Another study found that, for South Asian patients, nurturing and personal attention were seen as indicators of good care [30]. As such, patients preferred passive treatment, such as massage, not because they are “passive copers” but because passive treatments validate their sick role and the cultural norm of being nurtured when sick. Likewise, another article described the desire for passive care (*Amae*) in Japan to build trust (*Uchi*) before transitioning to active rehabilitation [61].

#### 3.2.4. Engaging Cultural Experts

Thirteen articles identified engaging cultural experts as crucial to culturally competent physical therapy [29,30,31,32,40,42,45,48,49,54,56,62,63]. This involved employing cultural liaisons, engaging community members, establishing advisory boards, and hiring providers with shared cultural identities.

For example, one study described the role of community liaisons and healthcare workers in the development of an intervention for Navajo cancer survivors [31]. They supported participant recruitment and addressed mistrust between Navajo individuals and Western medical professionals. Additionally, a community health partner assisted with intervention delivery, and a Navajo oncology nurse joined the research team. In another article on the development of a physical therapy program designed for the neonatal intensive care unit, they convened a standing advisory group of native Spanish-speaking, Latine members to review Spanish language study materials [48].

An article about a randomized controlled trial on culturally informed physical therapy described the intervention as a culmination of three years of engagement with Assyrian, Mandaean, and Vietnamese communities [40]. In another study with South Asian Canadians, providers conducted focus group discussions to learn first-person perspectives on how culture influences pain metaphors and care expectations [30].

#### 3.2.5. Intervention Selection and Adaptation

Another theme was the cultural adaptation of the intervention approach itself. Six articles adapted the intervention approach by incorporating culturally congruent activities [32,42,45,46,53,62], ten articles modified delivery methods [30,32,36,40,44,45,51,57,61,63], and four selected the intervention type based on cultural fit [29,32,43,45].

One study on the development of an intervention with the Dunghutti and Gumbangirr people in Australia to promote the musculoskeletal health of Indigenous people living in a rural community demonstrated the full range of intervention adaptations particularly well [45]. In consultation with community members, they selected a sports massage intervention because massage was considered culturally resonant and sports were perceived as a bridge between Aboriginal and non-Aboriginal people. Further, they incorporated introductory lectures on the traditional use of local bush medicines and massage oils, affirming the traditional healing system. Based on feedback from the advisory group, they modified the timing of the intervention to run in the morning and afternoons to accommodate the work contexts of Aboriginal health workers and participants. In the end, Elders in the community endorsed the interventions as “a collaborative, community-based project that affirms our traditional methods of healing and integrates these within an accredited course of study for our health workers and Elders”.

#### 3.2.6. Addressing Context Considerations

Another theme was accounting for contextual factors when culturally adapting interventions [30,33,36,39,45,48,52,54,63]. Authors addressed resource limitations by performing things such as providing low-cost or free exercises and accommodating small living spaces [27,31] and reducing barriers to care, such as transportation or childcare [31,45,54].

For Navajo cancer survivors, researchers designed home resistance exercises that used household items along with inexpensive equipment to ensure the intervention was sustainable [31]. They also noted that a free intervention with reimbursement for time and travel was essential to address the economic context of Navajo cancer survivors. Due to a high-density urban environment in Hong Kong, physiotherapists identified and modified exercises that could be performed in small living spaces [27].

#### 3.2.7. Explanatory Models of Health and Illness

Interventions are designed around explanatory models of health and illness: beliefs about what causes disease and how the body functions. A patient’s understanding may not align with a scientific model, which can impact communication, trust, and adherence. Several articles identified the misalignment of explanatory models of health and illness [28,29,33,51,52,55,56,62,63]. Specific examples included the following: Navajo models of healing as restoring balance versus fighting disease [28]; Zulu models of bewitching [63]; Chinese models of yin and yang energies [29]; Turkish models of the role of cold and damp in illness [52]; and Southeast European models of bioenergy healing [51]. None of the articles described how interventions were adapted to address incongruent explanatory models of health and illness.

#### 3.2.8. Provider Training

Nine articles described advancing providers’ cultural competence through training on cultural considerations and learning from patients [32,33,36,40,49,55,57,58,62]. Authors noted that training in cultural competence is the mechanism by which inequalities in health can be addressed [33,62]. They also noted that physical therapists receive little to no education on cultural competency [33] and lack the knowledge and skills to address cultural barriers in their practice [33,58]. Authors called for building cultural competency training into physical therapy education and professional development [40,58].

Several articles described specific trainings on cultural competency for practitioners. For instance, authors describe a three-day, pre-departure training on Inuit culture and life in Nunavik, followed by on-site training [36]. The authors noted that brief training was insufficient and recommended continuous cultural safety activities, including mentoring by Inuit workers and participation in community events.

## 4. Discussion

Research on the cultural adaptation of physical therapy interventions remains limited. There is a dearth of experimentally designed research, totaling five articles. Studies reported no significant differences in standard versus culturally adapted interventions on health literacy, pain knowledge, pain severity, pain interference, back pain, physical function, negative emotional state, quality of life, disability, pain beliefs, catastrophizing, and fear of movement. The range of intervention types under study was wide, to the extent that none have been tested more than once. There is also a lack of standardized procedures for culturally adapting physical therapy interventions. This makes it impossible to compare findings across studies based on distinct approaches to cultural adaptation. Further research is needed to improve reporting, clarify adaptation approaches, and identify which adaptations contribute to improved outcomes.

The experimentally designed research highlights several areas for further investigation. A culturally adapted physical therapy intervention that included a combination of group and individual sessions designed to address language, cultural, and access barriers showed greater impact on attendance, adherence, and pain-related suffering, but not on pain severity, function, interference, or emotional state. One study reported positive evidence for cultural mentorship on patient activation [39]. No studies reported negative outcomes associated with the cultural adaptation of interventions; however, this finding should be interpreted with caution given the potential for reporting bias.

Despite the limited amount of experimental evidence supporting the cultural adaptation of physical therapy, non-experimental research described a range of reported benefits of cultural adaptation, including perceived intervention benefit, clinical improvement, knowledge application, skill development, patient empowerment, cultural acceptability, cultural relevance, patient relevance, enhanced communication, intervention access, and understandability of intervention content. Adaptation strategies included language, content, and intervention adaptation; addressing norms, values, and context considerations; reconciling explanatory models of health and illness; engaging cultural experts; and providing training for providers. These represent types of cultural adaptation that could be considered by physical therapists and addressed in physical therapy curricula. At the same time, these non-experimentally designed studies do not allow for causal claims and should be interpreted as preliminary and with caution.

An established cultural adaptation framework provides a structure to advance and clarify a discussion about the state of the research on the cultural adaptation of physical therapy. The Purnell Model for Cultural Competence describes 12 cultural domains and provides a framework for considering cultural factors relevant to health care; it was also used by one study included in this review [33]. Following our synthesis of the included articles and identification of emergent themes, we used the Purnell Model as an interpretive framework in the discussion to contextualize the review findings. Examining areas of alignment and limited representation across the Purnell domains provides a structured way to consider strengths, gaps, and priorities for future research on the cultural adaptation of physical therapy. Six of the twelve domains were addressed in the reviewed articles and are described in the results section. They included the following domains: (1) Communication (language use, communication style, nonverbal behaviors); (2) Health Care Practices (beliefs about illness, treatment, pain, barriers to care); (3) Family Roles and Organization (family structure, child-rearing practices, views on relationships); (4) Overview/Heritage (personal background, migration history, living context, social conditions); (5) Health Care Practitioner Concepts (perceptions of and preferences about health care providers); and (6) Spirituality (religious beliefs, sources of meaning).

Two domains were addressed in a limited manner. The literature did not extensively address (7) Workforce Issues (work expectations, acculturation, practices). Notable exceptions included the adaptations of employment examples in intervention materials and accommodating work schedules. Work and employment are critical to rehabilitation processes and goals and should be further explored in research. There was also limited discussion of (8) Nutrition (food availability, meanings of food, dietary practices). The articles addressed fasting during Ramadan but little more. Again, this gap is important due to the foundational nature of nutrition in rehabilitation and its centrality in culture.

Four domains were not addressed in the articles. There was no discussion of (9) Biocultural Ecology (physical, genetic, and biological differences that affect health); (10) Death Rituals (beliefs and practices related to dying and mourning); (11) Pregnancy (beliefs and practices related to fertility, contraception, pregnancy, birth, and postpartum care); or (12) High-Risk Behaviors (behaviors that increase health risk, such as substance abuse and unsafe sexual practices).

The lack of research specific to pregnancy and high-risk behaviors is highly relevant for physical therapy. Pregnancy is a physical state that alters biological responses, such as ligament laxity, and requires specific physical therapy care. At the same time, it is culturally salient and variable across cultures. As such, it presents high potential for cultural incongruities related to appropriate physical therapy care and should be addressed in cultural adaptation practices, education, and future research.

High-risk behaviors are highly relevant to physical therapy, while simultaneously presenting in deeply cultural ways. There are practices that may not be familiar to practitioners and are not known to be harmful. For instance, betel quid (a chewable mixture of areca nut, betel leaf, slaked lime, and sometimes tobacco) use is practiced in many Asian and some other populations and has health consequences, including addiction, oral cancer, and respiratory and cardiovascular issues, but remains unfamiliar to many Western practitioners. It is critical that physical therapists are equipped to identify and address the range of high-risk behaviors likely to present in the diverse patient populations they serve. More specifically, high-risk health practices were not addressed in this literature. An example is burning for pain relief, which can lead to painful scarring and infection. The therapist must navigate the cultural salience and harmful effects of these types of health practices. There is a need to develop research- and practice-based clinical and educational guidance on how to navigate high-risk behaviors in cross-cultural physical therapy.

Another topic not addressed in the literature is that an intervention may be incompatible with the values, practices, or contexts of cultural groups. For instance, the reviewed literature showed that active rehabilitation approaches were poorly suited to some cultural groups, whereas passive treatments aligned with understandings of good medical care, how the body works, and the need for trust-building [30,51,61]. In these populations, an intervention based on active treatment, such as online physical therapy, may be incompatible. While adaptations are possible, the extent of modification required may alter the intervention so substantially that its core components, and thus its fidelity and efficacy, are compromised. Just three articles addressed how the intervention studied was selected. There is a need for a systematic approach to assess a base intervention’s pre-adaptation fit for cultural groups.

There are limitations to this review. As previously noted, the articles are heterogeneous in method, intervention, and outcomes. This limits comparability across studies. Second, reporting bias is possible; it may be that this body of research overestimates the success of adaptation processes, while underrepresenting failed ones. Third, the exclusion of non-English language publications presents a bias toward Global North approaches and potentially misses research in this area. The included studies span multiple countries and cultural contexts, and the extent to which adaptation strategies identified in one setting are transferable to other contexts is unclear. Lastly, the review was registered retrospectively; therefore, the timing of methodological decisions cannot be independently verified through a prospectively registered protocol.

## 5. Conclusions

This scoping review shows that cultural adaptation in physical therapy is an emerging but underdeveloped area of research. Few studies used experimental designs, and evidence of the effectiveness of culturally adapted interventions remains mixed. Existing studies reported favorable findings related to engagement, attendance, adherence, patient activation, and perceived relevance of care. Cultural adaptation extends beyond language and includes modifications to intervention content, delivery methods, communication strategies, culturally relevant examples and metaphors, provider training, and contextual factors that influence care. Important gaps remain, including limited attention to several cultural domains and a lack of systematic approaches for evaluating intervention fit, adaptation processes, and fidelity. Future research should examine the effectiveness of specific adaptation strategies and clarify when adaptation is warranted. A stronger evidence base could support more equitable and effective physical therapy for culturally and linguistically diverse populations.

## Figures and Tables

**Table 2:** Non-experimentally designed quantitative studies and qualitative studies on culturally adapted physical therapy.

Author	Target Population	Adaptation Framework	Methods	Main Finding
Non-experimentally designed quantitative studies				
Davis et al. 2018 [34]	Canada	RE-AIM implementation framework	Pre-post assessment and interviews (*n* = 72); 58 completed follow up data	A Danish education and exercise program for people with hip or knee osteoarthritis, GLA:D™, was successfully implemented in Canada with high patient satisfaction and similar improvements in pain and function as seen in Denmark.
Kaya Kara et al. 2024 [29]	Turkish children with chronic pain	Beaton cross-cultural adaptation guidelines	Delphi study (*n* = 38 experts, mothers and children)	PNE4Kids was culturally adapted for Turkish children through modifications to terminology, stories, metaphors, and visual content. Experts reached consensus on the relevance of information, feasibility of stories, and visual information.
Kenea et al. 2025 [42]	Stroke survivors in Ethiopia	Human-centered design with participatory co-creation	Pilot usability study (*n* = 10)	AdaptRehab VR was culturally contextualized through stakeholder co-creation, use of Afaan Oromoo, culturally relevant objects, and iterative modification of game content and difficulty. Patients and clinicians rated the system as easy to use and useful for rehabilitation.
Monroe et al. 2025 [32]	Latino adults with chronic neck or low back pain	Intervention Mapping-Adapt (IM-Adapt) and FRAME	Needs-assessment survey (*n* = 358) and a case series.	Systematic adaptation produced the GOALS/Metas intervention, with modifications to language, content, therapeutic exercises, materials, delivery, and treatment format. Pilot testing supported feasibility and acceptability of the culturally and linguistically adapted intervention.
Park et al. 2014 [43]	Ethnically diverse older adults in the U.S.	None	Descriptive cross-sectional survey (*n* = 281)	While doctors most often recommended non-aquatic exercise and physical therapy for chronic pain management in ethnically diverse older adults, only exercise (82.5%) was commonly used, with prayer being a significant factor for some ethnic groups (50.7%); other strategies, such as aromatherapy, acupuncture, and occupational therapy, were not frequently used (fewer than 2%).
Pascal et al. 2024 [44]	People poststroke at risk for falls in Guyana	None	Pretest–posttest pilot study (*n* = 20 enrolled; *n* = 19 analyzed)	A falls-prevention program was adapted to Guyana’s cultural context. Following the 8-week program, participants showed significant improvements in walking speed, sit-to-stand performance, concern about falling, and fall frequency.
Vindigni et al. 2004 [45]	Aboriginal people living in a rural community in Australia	Consultation and informal round table approach	Pre-post assessment and feedback form (*n* = 20)	A culturally tailored sports massage training program for Aboriginal Health Workers and community Elders was successfully implemented in rural Australia, demonstrating improvements in knowledge and competence in all training topics.
Mansoor Rahman et al. 2026 [46]	Individuals with cerebral palsy in India	No cultural adaptation framework	Pre-post intervention (*n* = 36)	Traditional Indian folk games were culturally adapted by modifying rules, equipment, environment, time, and tasks. The intervention was feasible and culturally relevant, with significant improvements in handgrip strength, skinfold measures, and physical, emotional, and school HRQoL.
Qualitative studies				
Bostick et al. 2021 [30]	South Asian	None	Focus Groups (*n* = 14)	Focus groups with people of South Asian descent living with pain revealed culturally relevant themes like communication barriers, prioritizing function over pain intensity, valuing personalized care, and traditional healing beliefs, highlighting the need for culturally adapted pain management.
Colón-Semenza et al. 2024 [35]	Latino/a/e with Parkinson disease in the U.S.	Social ecological model	Phenomenological study; 5 focus groups (*n* = 25)	Latino/a/e participants identified barriers and facilitators to exercise and PT, including less specific exercise knowledge, family influences, financial barriers, prior emotional experiences, group-exercise experiences, access, and cultural beliefs. Findings support culturally and linguistically appropriate physical therapy, inclusion of family and caregivers, and improved access to affordable, culturally tailored exercise services.
De Heer et al. 2019 [31]	Navajo	Intervention Mapping	Interviews and focus groups (*n* = 53)	Using a structured process called Intervention Mapping, researchers developed a culturally sensitive physical activity program for Navajo cancer survivors, incorporating their preferences, values, and environmental considerations to improve participation and health outcomes.
Dressler and Pils 2009 [47]	Migrant and ethnic minority patients	None	Interviews (*n* = 28)	The study examined how the staff of a post-accident inpatient rehabilitation center in Austria perceived cross-cultural communication with migrant and ethnic minority patients. They focused on cultural adaptation topics related to language barriers, religious considerations, gender considerations, cultural differences in rehabilitation goals, pain expression, and the need for institutional support.
McCarty et al. 2024 [48]	Spanish language speakers	None	Document analysis, review of session facilitation	Bilingual physical therapy students, with feedback from a Latine community review board, translated and culturally adapted parent education materials for a program supporting maternal mental health and infant development in the Neonatal Intensive Care Unit, increasing accessibility for Spanish-speaking families.
Najem et al. 2025 [28]	Lebanese adults with chronic low back pain	Ecological Validity Model (EVM) and Barrera cultural adaptation model	Focus groups (*n* = 20)	Co-design identified the need to culturally adapt PNE across language, metaphors, content, context, and delivery. Religious beliefs and culturally specific understandings of pain were particularly important. Participants favored interactive, therapist-delivered education and emphasized the need for ongoing refinement of materials to support culturally responsive pain care.
Nelson et al. 2025 [49]	Aboriginal and Torres Strait Islander adults	None	Semi-structured interviews (*n* = 12)	Culturally safe physiotherapy involved building trust through yarning, acknowledging and respecting culture, creating culturally safe clinical environments, and improving physiotherapists’ cultural learning. Respectful communication and consent before hands-on treatment were particularly important for comfort, trust, and engagement.
Orhan et al. 2019 [50]	Turkish patients with chronic pain	None	Delphi study s(*n* = 10) (8 experts, 2 patients)	Researchers successfully adapted Pain Neuroscience Education materials for first-generation Turkish patients with chronic pain by incorporating feedback from experts and patients, resulting in culturally sensitive resources to improve understanding and management of chronic pain.
Scheermesser et al. 2012 [51]	Southeast Europeans	None	In-depth interviews (*n* = 13)	This study in Switzerland found that Southeast European patients with low back pain preferred passive coping strategies and had difficulty understanding active rehabilitation approaches, highlighting the need for culturally sensitive communication, interpreters, early intervention, and focus on return-to-work strategies.
Sturkenboom et al. 2007 [52]	Turkish or Moroccan female immigrants and family members	None	In-depth interviews (*n* = 9)	This study found that family members of female immigrants with chronic pain had limited understanding of rehabilitation and their role in it, emphasizing the need to involve families and develop culturally sensitive strategies to improve rehabilitation outcomes.
Wickford et al. 2008 [53]	Afghans	None	In-depth interviews (*n* = 13); participant observation, photography	This qualitative study examined the Rehabilitation of Afghans with Disability program. Treatment was primarily passive rather than active because patients found exercise “boring”, and passive treatments aligned with cultural beliefs. Key cultural considerations were noted, such as gender, modesty, and fatalism.
Yeowell, G. et al. 2010 [54]	Pakistani, Muslim female patients	None	Interviews (*n* = 6)	This qualitative study explored the physiotherapy needs of female Pakistani patients in England and found that cultural factors such as gender preferences, communication barriers, appointment length, and understanding of exercise affected their experiences, highlighting a need for culturally competent care to improve compliance and address health disparities.

**Table 3:** Non-empirical articles on culturally adapted physical therapy (review articles, discussions, case examples, and editorials).

Author	Target Population	Adaptation Framework	Main Finding
Al Zoubi et al. 2023 [27]	Hong Kong context	ADAPTE framework; review with the AGREE II tool	Researchers in Hong Kong adapted international guidelines for knee and hip osteoarthritis physiotherapy management using the ADAPTE framework, resulting in culturally and clinically relevant recommendations for local practitioners, including a strong emphasis on land and aquatic exercise, education, and self-management.
Black et al. 2002 [33]	General	Purnell’s Cultural Model	This article emphasizes the critical need for physical therapists to develop cultural competence to effectively interact with diverse clients. It highlights that cultural differences exist in every clinical encounter, even beyond obvious primary characteristics like race and ethnicity.
Brady et al. 2016 [55]	Australia	None	This excerpt highlights the growing cultural diversity in Australia, fueled by increased migration from non-English-speaking countries. This diversity presents a challenge for the healthcare system to provide culturally responsive care that considers social, economic, and cultural factors influencing health disparities, recommending cross-cultural skills and training for all levels of physiotherapy students.
Hunt et al. 2007 [56]	General	None	This article emphasizes the growing importance of cultural awareness in physiotherapy due to increasing cultural diversity in Western nations. It highlights that cultural backgrounds influence how people perceive and experience health, illness, and disability, shaping their expectations of healthcare providers and their therapeutic goals.
Hutting et al. 2026 [57]	Diverse populations with chronic musculoskeletal conditions	Mature culturally sensitive biopsychosocial model	Proposes an expanded biopsychosocial model in which culture has an overarching influence across biological, psychological, and social domains. The model emphasizes culturally responsive communication, collaborative goal setting, consideration of beliefs, expectations, life goals, and social/economic/spiritual functioning, and culturally tailored self-management support within musculoskeletal physiotherapy.
Kawathekar & Campbell 2023 [58]	General	None	This is a very brief editorial broadly about cultural competence in physical therapy. It recommends that the cultural competence process start in entry-level physiotherapy studies.
Monroe et al. 2025 [59]	Latino adults with chronic spine pain in the U.S.	Intervention logic model for culturally tailored psychologically informed physical therapy (PIPT)	Proposes a framework for culturally tailoring PIPT while retaining four essential treatment elements: cognitive skill training, aerobic activity, therapeutic exercise, and graded functional movement. Sociocultural factors should guide adaptation of therapeutic goals, language, content, delivery, and outcome assessment to improve fit for specific populations.
Norris & Allotey 2008 [60]	General	None	This article argues that physiotherapists must recognize their profession’s inherent cultural biases and actively cultivate cultural competence through awareness, acceptance, and collaboration to provide effective care in diverse contexts.
Odawara et al. 2005 [61]	Japanese patients	None	Analysis of two Japanese occupational therapy case studies reveals how culturally relevant therapeutic interventions, such as cooking linked to traditional female roles and building local social relationships, can enhance patient engagement and therapeutic outcomes.
Ouellet et al. 2022 [36]	Nunavimmiut	None	Developing a new rehabilitation program in the Inuit community of Nunavik presented numerous challenges for occupational and physical therapists, including communication barriers, cultural differences, cross-cultural interactions, population-specific knowledge, use of culturally safe assessment tools, and professional isolation, highlighting the need for culturally adapted practices and reflective approaches to improve service quality.
Ratima et al. 2006 [62]	Mäori	Framework based on: Guidelines for Cultural Competence in Physiotherapy Education and Practice in Aotearoa/New Zealand	New Zealand’s Health Practitioners Competency Assurance Act provides a valuable opportunity for physiotherapists to enhance cultural competence and address health inequalities, particularly for Māori. By understanding and implementing cultural competence standards, physiotherapists can contribute to a more equitable and effective healthcare system that respects the Treaty of Waitangi and acknowledges the impact of ethnicity on health outcomes.
Reis et al. 2022 [63]	General	None	To optimize pain management in diverse populations, clinicians must consider cultural influences on pain perception and tailor education and exercise programs accordingly, using culturally appropriate language, metaphors, examples, and activities.

## Data Availability

No new data were created or analyzed in this study. Data sharing is not applicable to this article.

## Supplementary Materials

The following supporting information can be downloaded at https://www.mdpi.com/article/10.3390/healthcare14193317/s1.
